# The Impact of Silver Diamine Fluoride Only or Simultaneously With Potassium Iodide Treatment on the Bond Durability of Resin Composite Material on Primary Teeth

**DOI:** 10.7759/cureus.57064

**Published:** 2024-03-27

**Authors:** Nesreen Y Mohammed, Dina M Abdel-Ghany, Naoufel Ben Hamadi, Sadin Özdemir, Zeliha Selamoglu, Gabriel Plavan, Fehmi Boufahja, Dalia M Elassar

**Affiliations:** 1 Dental Biomaterials Department, Faculty of Dental Medicine for Girls, Al-Azhar University, Cairo, EGY; 2 Pediatric Dentistry and Oral Public Health Department, October 6 University, Giza, EGY; 3 Chemistry Department, College of Science, Imam Mohammad Ibn Saud Islamic University (IMSIU), Riyadh, SAU; 4 Food Processing Programme, Technical Science Vocational School, Mersin University, Mersin, TUR; 5 Medical Biology Department, Medicine Faculty, Nigde Omer Halisdemir University, Nigde, TUR; 6 Biology Department, Faculty of Biology, Alexandru Ioan Cuza University, Iasi, ROU; 7 Biology Department, College of Science, Imam Mohammad Ibn Saud Islamic University (IMSIU), Riyadh, SAU; 8 Operative Dentistry Department, Faculty of Dental Medicine for Girls, Al-Azhar University, Cairo, EGY

**Keywords:** resin composite, universal adhesive, primary teeth, bond durability, potassium iodide, silver diamine fluoride

## Abstract

We aim to evaluate the bond strength between resin composite and primary demineralized dentin, pretreated with silver diamine fluoride (SDF) and simultaneous SDF with potassium iodide (KI) after thermal aging. In this in vitro study, human carious-free primary molars were randomly assigned into three groups and prepared by exposing the superficial dentin. The primary dentin of each molar was demineralized. The first group (the control) received saline treatment before bond application. SDF was pretreated for the second group, whereas SDF and KI were used for the third. After that, the pretreated dentin was immediately built with resin composite bonded with a universal adhesive and kept wet for 24 hours. Then, the pretreated molars were prepared into beam specimens for microtensile bond strength (µTBS), 16 for each group, and subjected to thermal aging. Lastly, they were tested using a universal testing machine, and the resulting data were analyzed using one-way analysis of variance (ANOVA) followed by Tukey's post hoc test. It was found that the SDF-KI group had a significant difference with both the control and SDF groups (p < 0.05), while the control and SDF groups showed no significant differences (p = 0.310). The SDF-KI group had the highest mean value of 11.73 ± 4.39 megapascal (MPa). In contrast, the control group had the lowest mean value of 9.31 ± 3.41 MPa. Post hoc pairwise comparison results showed that SDF-KI pretreatment had a significantly higher strength value than the control and SDF groups. Pretreatment of demineralized primary dentin with SDF-KI does not negatively affect the immediate loading of resin composite. However, under the limitation of this study, KI application after SDF pretreatment is recommended to enhance the bond's durability of resin composite to demineralized dentin.

## Introduction

The importance of silver diamine fluoride (SDF) application as minimally invasive and non-aerosolizing management has greatly increased especially in cases of active caries [1]. Furthermore, it may be a promising strategy to manage dental caries with children in general or those who have special needs [2]. SDF with a concentration of 38% was approved by the Food and Drug Administration (FDA) in 2016 as a substance that stops the process of tooth decay in a noninvasive approach to preserving tooth structure [3]. It is an antibacterial and cost-effective alkaline solution of diamine silver and fluoride ions. Diamine silver ions are formulated in a complex with two ammonia molecules bonded to a silver ion, making it more stable and less oxidative than silver ions alone. Furthermore, fluoride ions are present at a higher concentration, which is 44,800 ppm, than in any fluoride agent. Such a combination of ions in that alkaline solution has a synergistic effect in arresting dentine caries, which makes SDF different from other fluoride agents [4]. Studies have also reported that SDF was better than fluoride varnish in arresting caries in primary teeth and comparable to or better than glass ionomer cement [5].

Despite the previously mentioned advantages of the SDF application, there are limitations to its use. For example, the SDF application's black stain may interfere with aesthetics. Additionally, the masticatory function of the treated SDF cavitated teeth may not be improved because they are not restored. Saturated potassium iodide (KI) is one of the solutions introduced to decrease the staining effect caused by SDF application, resulting in a white silver iodide precipitate; it masks the color of SDF (38%) by 100% due to forming a white precipitate [6]. Moreover, restoring a cavitated tooth treated with SDF improves chewing ability and seals the tooth against bacteria, protecting it from further recurrent caries [7]. This technique is called the Silver Modified Atraumatic Restorative Technique (SMART) if resin composite or resin-modified glass ionomer (RMGI) is used as restorative materials [8]. However, the bond strength of direct restorative materials especially resin composites to SDF pretreated dentin is still uncertain. Studies show that the effect of SDF on the bonding of adhesives appears to be dependent on the protocol of application [9].

Many research investigations have found inconsistencies in data regarding the bond strength of resin composite restored teeth previously treated with SDF or SDF-KI. Although the rinsing step has eliminated the negative effect of SDF application, SDF applied previously to the adhesive system significantly impaired the bond strength to dentin mainly when not rinsed [10]. Others showed that immediate loading after SDF and SDF-KI treatment decreased the adhesion of resin composite restoration [11]. The silver particles extend into the dentinal tubules, causing their total or partial obstruction and interfering with micromechanical retention, the infiltration of adhesive, and thus less hybrid layer formation.

In vitro, aging methods have been considered a must in any laboratory adhesion study, as they may help researchers better understand and predict the performance of adhesive systems concerning bonding degradation. Various methods have been introduced to check bond durability, including the application of thermal and mechanical cycling [12]. However, ideal vitro studies that were performed did not explain how the materials might behave inside the oral cavity [13]. Thermocycling is one of these methods that is preferred because it simulates moisture and temperature changes in oral conditions. Although the aging of the bond strength of SDF and SDF-KI was investigated at a frequency of 1,000 thermal cycles on permanent sound dentin using total-etch and universal adhesives [9], it was reported that such used cycles did not cause any decrease in the bond strength and, hence, might not represent bond durability [14]. There is controversy regarding the effect of SDF and SDF-KI application on the initial bond strength and bond durability of dentin-to-resin composites. This is likely due to the large range of differences between the studies or the lack of investigation of bond durability [15]. Also, there was a lack of studies that examined the bond durability of resin composite restoration to dentin surfaces pretreated with SDF-KI.

Therefore, this study aimed to investigate the impact of SDF and SDF-KI on bond durability between resin composite and demineralized primary dentin using universal adhesive and evaluated the failed interfaces under laboratory conditions. The study was conducted to accept or reject the null hypotheses that there was no difference in the bond durability of resin-based composite (RBC) on demineralized primary dentin pretreated with SDF or SDF-KI compared to primary demineralized dentin without pretreatments (control). However, under the limitation of this study, KI application after SDF pretreatment is recommended to enhance the bond's durability of resin composite to demineralized dentin.

## Materials and methods

Materials used in the study

**Figure 1:**
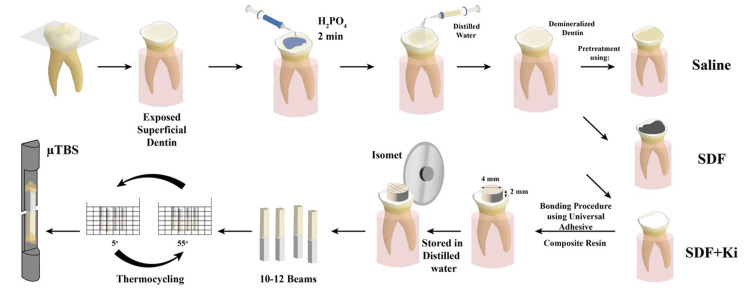
Graphical abstract of the procedures of beam specimen preparation for µTBS µTBS: microtensile bond strength, SDF: silver diamine fluoride, SDF+Ki: silver diamine fluoride with potassium iodide

The materials used in this study are illustrated in Table 1. A graphical abstract was demonstrated to show the procedures of the preparation of beam specimens to be evaluated for microtensile bond strength (µTBS) (Figure 1).

**Table 1:** Materials used in the study SDF: silver diamine fluoride, KI: potassium iodide, 10-MDP: 10-methacryloyloxydecyl dihydrogen phosphate, HEMA: 2-hydroxyethyl methacrylate

Name of materials	Composition/specification	Manufacturer
SDF	38% w/w active ingredients in deionized water.	TOOTHMATE, Egypt
KI	Potassium iodide in deionized water	TOOTHMATE, Egypt
All bond universal adhesive	10-MDP, dimethacrylate resins, HEMA, ethanol, water, initiators, colloidal silica	BISCO Inc., USA
Resin composite (Xs-Fil)	Nano-hybrid resin composite	B&E, Korea
Fine each	Phosphoric acid (H_3_PO_4_) 37%	Spident, Korea

Experimental design

This laboratory experimental study was conducted at the Faculty of Dental Medicine for Girls of Al-Azhar University, Cairo, Egypt. It was approved by the Ethical Research Committee, Faculty of Dental Medicine for Girls, Al-Azhar University, Cairo, Egypt (approval code: REC-PD-23-12). Before starting the experimental study, the number of specimens required in each group was determined after a power calculation according to the data obtained [16]. In that study, the microtensile bond strength (µTBS) in the SDF group was 56.6 ± 29.8 µTBS and that in the SDF-KI group was 100.58 ± 41.53 µTBS, with a large effect size (f = 0.832). Based on the study, 48 specimens were divided into three groups. Sixteen beams in each group were determined to provide 80% power for independent samples t-tests at the level of 5% significance and a confidence interval of 95% using G. Power 3.19.2 software.

Fifteen human deciduous carious-free anonymous molars (lower second primary molars without any hypoplastic spots, deformation, or restoration) were collected by extraction due to their delayed shedding that interfered with the eruption of underlying premolars. Consent was taken by the pedodontics department during the extraction. They were obtained from the Department of Pediatric Dentistry, October 6 University, Cairo, Egypt. The teeth were properly cleaned under running tap water with a soft toothbrush with non-fluoridated paste to remove debris, blood, and plaque. They were disinfected using aqueous chloramine and stored in a thymol solution at 4°C.

Tooth preparation and bonding procedures

Teeth were randomly divided into three groups depending on the received pretreatment. A cylindrical Teflon mold (2 cm diameter and 2 cm height) was used to obtain acrylic resin blocks. Each primary molar was embedded while the acrylic was still soft. After the setting of the acrylic resin, the mold was removed. Afterward, a slow-speed cutting machine was used to remove the occlusal enamel of all primary teeth using water as a coolant. The enamel was completely removed by examining the dentin surfaces under a stereomicroscope at 30× (Eclipse MA-100, Nikon, Tokyo, Japan). The primary dentin of teeth was demineralized using phosphoric acid gel 37% (each fine) (Spident, Korea). It was applied on primary dentin for two minutes, followed by washing with a copious amount of water for an additional two minutes.

These demineralized dentin teeth were categorized into three groups based on the pretreatment they received. For the control group, demineralized dentin did not receive any pretreatment (only treated with saline). In the SDF group, only SDF was applied as pretreatment on demineralized dentin. In the SDF-KI group, SDF-KI was applied simultaneously on demineralized dentin.

In the SDF group, two drops (0.1 mL) of SDF were applied directly to the demineralized dentin with a microbrush for one minute with agitation. The excess material was removed using gauze. After applying KI to SDF previously added to the SDF-KI group, a white precipitate was formed. The addition should be continued until the white precipitate is no longer visible. The pretreated dentin surfaces in the SDF, and SDF-KI were not rinsed.

After that, a universal adhesive (All-Bond Universal, BISCO, USA) was applied on previously pretreated primary dentin in two layers as per the manufacturer's instruction. Then, they were cured by a light-emitting diode (LED) light unit (RTA, MiniS Curing Light, China) device with 1,000 mW/cm^2^ intensity and an effective wavelength of 420-480 nm for 15 seconds. The LED unit device was monitored after every six shots using a radiometer (Cure Rite Efos, model 8000, Efos Inc., Mississauga, Ontario, Canada). The radiometer was used to give a relative indication of the intensity output of curing light. Dentin specimens were built post-pretreatment with resin composite (nano-hybrid resin composite) (B&E, Korea) with the aid of a Teflon mold (2 diameter × 4 height, mm^2^) for standardization. It was added in 4 mm increments and cured for 40 seconds individually. After that, specimens were submerged in distilled water at a temperature of 37°C for 24 hours.

Microtensile bond strength (µTBS) testing

Beam Specimen Preparation

Longitudinal sectioning of the earlier restored pretreated dentin was performed to obtain beams with 1 mm^2^ of bonding area. Each beam was composed of resin composite and dentin with adhesive at the interface. They were obtained by mounting a pretreated tooth in the gripping attachment, which was serially sectioned, using a 0.3 mm thick diamond-coated disc of cutting machine (IsoMet 4000, Buehler, Germany) under copious coolant. This resulted in 10-12 beams with 1 mm^2^ of bonding area per sectioned restored tooth. A digital caliper (Total Tools, Malaysia) was used to check the dimensions of all beams. Any fractured beam or not within the required dimension was discarded. Also, any specimen near to pulp horn in length was considered not suitable for µTBS evaluation. Later, these beams were stored in distilled water at room temperature in tight-sealed plastic cones labeled according to group.

Bond Durability Measurements Using Microtensile Bond Strength (µTBS)

The resultant beams were subjected to thermal cycles: 5,000 cycles at 5°C and 55°C with a dowel time of 30 seconds in the thermocycling device (SD mechatronic thermocycler, Germany). Afterward, only 16 still-bonded beams were selected randomly from each group to determine the bonding performance. Finally, each beam was fixed using cyanoacrylate-based adhesive in the testing jig and mounted in the universal testing machine (Model 3345, Instron, England). The beams were stressed in tension at a crosshead speed of 0.5 mm/minute until the bonding failure of the specimen occurred. Bond strength was calculated in megapascal (MPa).

Evaluation of failure mode

The failure mode was assessed quantitatively using a stereomicroscope (MA-100, Nikon, Tokyo, Japan) with 30× magnification to examine the failure mode of the samples. De-bonded surfaces were categorized according to the mode of failure into one of the following three groups: (1) cohesive (completely in dentin or resin composite substrate), (2) adhesive (in dentin resin interface), and (3) mixed (combination of adhesive and cohesive failure).

Statistical analyses

Data were collected and presented in tables and figures. Data normalities were checked using the Shapiro-Wilk test to check whether the data were parametric or nonparametric. Accordingly, microtensile bond strength was parametric, and mode of failure was nonparametric. Nonparametric data are represented as frequency and percentage values and were analyzed using Fisher's exact test. Parametric data are represented as mean and standard deviation. The homogeneity of variances was examined using Levene's test. Differences between the control, SDF-KI, and SDF groups were analyzed using the one-way analysis of variance (ANOVA), followed by Tukey's post hoc test at a significance level of p < 0.05. Statistical analysis was performed with R statistical analysis software version 4.1.3 for Windows (R Foundation for Statistical Computing, Vienna, Austria).

## Results

Table 2 shows mean and standard deviation values for intergroup comparison of µTBS. It showed that the SDF-KI group had a significant difference with both the control and SDF groups (p < 0.001), while there was no statistically significant difference between the control and SDF groups (p= 0.310). The highest mean value was found in the SDF-KI group (26.39 ± 2.93 MPa), followed by the SDF group (11.73 ± 4.39 MPa), while the lowest mean value was found in the control group (9.31 ± 3.41 MPa). Post hoc pairwise comparison results of the SDF-KI group showed a significantly higher strength value than that of the SDF group. Moreover, the result of the former showed a significantly higher strength value than the control group.

**Table 2:** Mean and standard deviation values for intergroup comparison of µTBS Different superscript letters indicate a statistically significant difference within the same horizontal row. *Significant (p < 0.05) µTBS: microtensile bond strength, MPa: megapascal, SD: standard deviation, SDF: silver diamine fluoride, KI: potassium iodide

Microtensile bond strength (MPa) (mean ± SD)			F-value	p-value
Control	SDF-KI	SDF		
9.31 ± 3.41^B^	26.39 ± 2.93^A^	11.73 ± 4.39^B^	64.89	<0.001*

**Figure 2:**
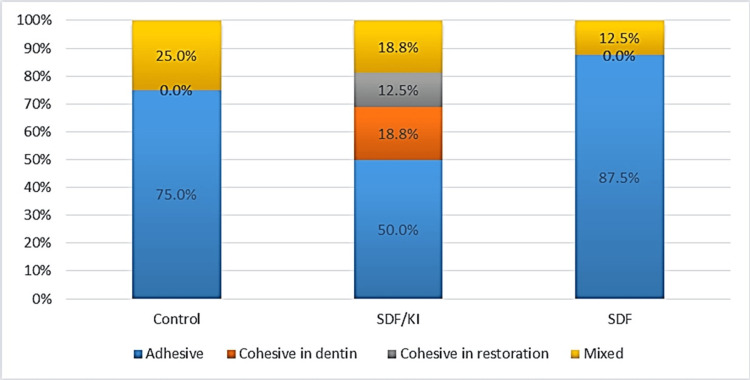
Stacked bar chart showing failure modes in different groups SDF: silver diamine fluoride, KI: potassium iodide

**Figure 3:**
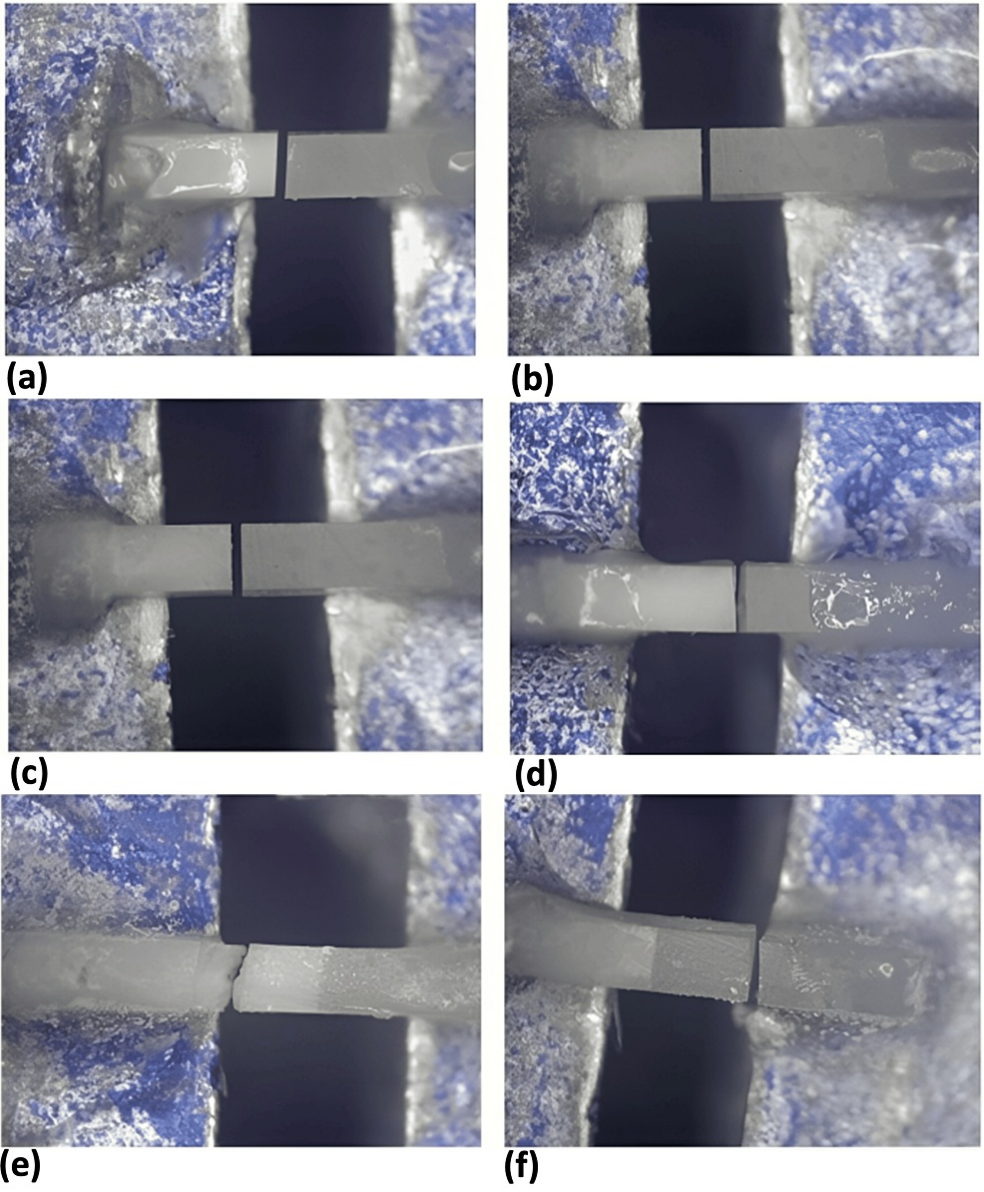
Stereomicroscope photos at 30× showing fracture mode of representative samples of each group a: Adhesive failure in the control group. b: Adhesive failure in the SDF group. c: Adhesive failure in the SDF-KI group. d: Mixed failure in the SDF-KI group. e: Cohesive failure in the dentin of the SDF-KI group. f: Cohesive failure in the resin composite of the SDF-KI group. SDF: silver diamine fluoride, KI: potassium iodide

The quantitative distribution of failure modes is presented in Figure 2. The most common mode of failure was adhesive in the control and SDF groups (Figures 2, 3a, 3b). However, half the samples of the SDF-KI group also failed adhesively (Figures 2, 3c). Also, for the latter group, the failure mode that came in second place was the mixed type (Figure 3d). Cohesive failure was also observed in a few samples in dentin (Figure 3e) and resin composite (Figure 3f). Nevertheless, the difference between the tested groups was not statistically significant using the Kruskal-Wallis test (p = 0.081).

## Discussion

The objective of this study was to evaluate the effect of SDF and SDF-KI pretreatment on bond durability between demineralized primary dentin and resin composite after thermal aging. The null hypothesis, which stated that there was no significant difference found in the bond durability of resin-based composite (RBC) to demineralized primary dentin using SDF or SDF-KI pretreatment compared to the control group, was partially rejected. As the bond durability after using SDF-KI was statistically significant than the SDF and the control groups, this part of the hypothesis was accepted. While the SDF group results were insignificant to the control one, it was rejected.

Phosphoric acid gel 37% was used in the present study to create affected dentin due to its ability to be applied for small areas as needed. Additionally, it is quick and effective in providing adequate demineralization. A concentration of 37% phosphoric acid gel was also used because it has been shown to produce a similar appearance of a smooth surface natural incipient lesion by application for two minutes [17].

A commercial 38% SDF solution that contains 44,800 ppm fluoride was chosen for the present study according to Fung et al. [18] who concluded that 38% SDF was statistically significant in caries prevention and arrest in comparison to 12% SDF in primary teeth. Laboratory studies found that SDF has a significant antimicrobial effect against cariogenic biofilm. Moreover, SDF was reported to possess a remineralizing and rehardening effect [19].

In the present study, we used thermocycling, one of the most common methods of artificial aging applied to investigate bond durability by promoting chemical degradation [20]. We followed the temperature range of 5°C-55°C defined in ISO/TS 11405, with a dwell time of 30 seconds. Additionally, 5,000 cycles used in the present study were reported to cause a decline in bond strength and, hence, bond durability [21]. On the other hand, the microtensile bond test is a very useful tool for bonding evaluation. It is conservative in sample teeth collection, more reliable, and easier in comparison between a variety of substrates and areas in the same tooth, and it has a more uniform loading stress distribution over a smaller bonded area [22].

In the current study, there was a statistically significant decrease in bonding performance in the SDF group compared to the SDF-KI group. It is possible that such a decrease in bond strength was due to the precipitation of silver deposits and silver oxide, which cause coagulation of exposed denatured collagen fibrils [23]. Additionally, silver particles extend into the dentinal tubules, causing their total or partial obstruction and interfering with micromechanical retention, infiltration of adhesive, and, thus, less hybrid layer formation [24]. This agrees with the study by Harnirattisai et al. [25], who demonstrated lower bond strengths of adhesive resins to amalgam-stained dentin. Moreover, this result was consistent with that of Markham et al. [26], who found that the stability of dentin bond to the universal adhesive in self-etch mode was reduced with SDF application. Also, our result agreed with that of Ko et al. [27], who found a decline in bond durability after 5,000 and 10,000 thermal cycles of the 38%SDF group.

However, the current results correspondingly showed a reduction in the bonding performance of the control group, which had the lowest value. This might be due to the demineralization process that was performed before the bonding procedures. Such a process made the dentin substrate devoid of hydroxyapatite crystals. One of the functions of phosphate ester monomer (10-MDP), the main component of universal adhesive, is the ability to bond chemically with the tooth minerals, increasing bond strength [28]. Mild etching of universal adhesives can preserve residual hydroxyapatite crystals around collagen fibrils, which results in improved bond stability. Additionally, the method of creation that affected dentin used in this study might increase the fluid flow through opened dentinal tubules, specifically in the exposed superficial dentin. This increases the susceptibility of the bonding interface to hydrolytic degradation, resulting in weakened long-term dentin bond strength [29].

In the SDF-KI group, statistically significant bonding performance was observed, surpassing that of both the SDF and control groups. This might be explained by the reaction between KI and SDF application. KI stimulates the production of silver iodide precipitate into the demineralized dentin substrate. It may block the dentinal tubules, reducing fluid flow and improving bonding performance [30]. By applying a universal adhesive, acidic monomer, 10-MDP reacts chemically through the ionic bond with the previously formed precipitate, increasing the bond strength. Moreover, the carboxylic group of HEMA-containing universal adhesive may bond to the formed precipitate in the dentinal tubules, thus creating a stronger bond [16]. During thermal aging, the formed precipitate might increase the resistance to hydrolytic degradation in this in vitro study. Our results agreed with those of Selvaraj et al. [30]. This was in contrast with the results of the study by Farahat et al. [9], who observed a decrease in bond strength in the SDF-KI group after aging, but it was not statistically significant. This might be due to the differences in SDF-KI application protocol in addition to dentin substrate conditions. Also, our results disagreed with Koizumi et al. [11]. However, this could be because the previous study used sound dentin as the substrate.

On the other hand, the failure mode of most specimens in the existing study was adhesive and mixed failure. Cohesive failure was observed only in the SDF-KI group in dentin or resin composite. The high bonding performance associated with this group might be correlated with this observation. In contrast, some studies explain cohesive failure by microcracks formed during specimen preparation and the material's fragility [15]. This finding is consistent with the fact that using SDF solution on dentin surfaces resulted in adhesive failure as the predominant mode of failure [27].

This study has several limitations. First, bonding durability was investigated only after thermocycling without an immediate bonding test. Second, the effect of bonding performance was tested without examination of remineralization. Although the current study sheds light on the remineralization effect of SDF and SDF-KI on demineralized dentin, further research is necessary to study the effect of bonding performance on a microscopic level and elemental analysis.

## Conclusions

Under the limitations of the current study, pretreatment of demineralized primary dentin with SDF and KI did not negatively affect the bonding performance of immediately loading resin composite. Besides, it appears that using SDF followed by KI application had a positive effect on the bond durability of immediate loading of resin composite to the demineralized dentin after thermal aging.
